# Metabolic Dysregulations and Epigenetics: A Bidirectional Interplay that Drives Tumor Progression

**DOI:** 10.3390/cells8080798

**Published:** 2019-07-30

**Authors:** Fabiana Crispo, Valentina Condelli, Silvia Lepore, Tiziana Notarangelo, Alessandro Sgambato, Franca Esposito, Francesca Maddalena, Matteo Landriscina

**Affiliations:** 1 Laboratory of Pre-Clinical and Translational Research, IRCCS, Referral Cancer Center of Basilicata, 85028 Rionero in Vulture, PZ, Italy; 2 Department of Molecular Medicine and Medical Biotechnology, University of Naples Federico II, 80131 Naples, Italy; 3 Medical Oncology Unit, Department of Medical and Surgical Sciences, University of Foggia, 71100 Foggia, Italy

**Keywords:** metabolism, epigenetics, crosstalk, cancer

## Abstract

Cancer has been considered, for a long time, a genetic disease where mutations in key regulatory genes drive tumor initiation, growth, metastasis, and drug resistance. Instead, the advent of high-throughput technologies has revolutionized cancer research, allowing to investigate molecular alterations at multiple levels, including genome, epigenome, transcriptome, proteome, and metabolome and showing the multifaceted aspects of this disease. The multi-omics approaches revealed an intricate molecular landscape where different cellular functions are interconnected and cooperatively contribute to shaping the malignant phenotype. Recent evidence has brought to light how metabolism and epigenetics are highly intertwined, and their aberrant crosstalk can contribute to tumorigenesis. The oncogene-driven metabolic plasticity of tumor cells supports the energetic and anabolic demands of proliferative tumor programs and secondary can alter the epigenetic landscape via modulating the production and/or the activity of epigenetic metabolites. Conversely, epigenetic mechanisms can regulate the expression of metabolic genes, thereby altering the metabolome, eliciting adaptive responses to rapidly changing environmental conditions, and sustaining malignant cell survival and progression in hostile niches. Thus, cancer cells take advantage of the epigenetics-metabolism crosstalk to acquire aggressive traits, promote cell proliferation, metastasis, and pluripotency, and shape tumor microenvironment. Understanding this bidirectional relationship is crucial to identify potential novel molecular targets for the implementation of robust anti-cancer therapeutic strategies.

## 1. Introduction

Cellular metabolic alterations have been recognized as a crucial hallmark of cancer due to their numerous implications in cancer-promoting signals. In their rapid and uncontrolled proliferation, solid tumors generate hostile microenvironments because the tumor mass can outgrow the existing tissue vascularization, thus limiting cellular access to oxygen (O_2_) and nutrients. Although angiogenesis is constantly stimulated during cancer progression, tumor blood vessel system is disorganized and leaky. As a consequence, many solid tumors grow in a hypoxic environment, which increases intracellular reactive oxygen (ROS) and nitrogen species (RNS) generation and causes endoplasmic reticulum (ER) stress [1]. Additionally, in these stressful and dynamic microenvironments, the concentrations of crucial nutrients, such as glucose, glutamine, and O_2_, have a spatially and temporally heterogeneous distribution [1] undermining the survival of cells in the tumor core. Thus, tumor cells promote adaptive metabolic reprogramming strategies to improve their fitness, support a selective proliferative advantage, and sustain new redox homeostasis to counterbalance the hypoxic/oxidative stress of dynamic tumor microenvironments [2,3,4,5,6,7,8].

Generally, the non-cancer cell metabolic program relies on ATP production to satisfy energetic requirements and maintain homeostatic processes. In contrast, highly proliferating cancer cells require not only enough energy to sustain cell replication, but also neo-synthetized macromolecules for rapid growth demand and maintenance of redox balance in response to intensive production of toxic reactive species. The growth and survival of tumor cells are fundamentally dependent upon the generation of metabolic adaptive solutions that meet these requirements. Thus, cancer cells implement various metabolic strategies to coordinate their core functions, such as bioenergetics, anabolic biosynthesis, and appropriate redox balance [3]. In such a context, the major biochemical pathways altered in cancer cells are glycolysis and tricarboxylic acid (TCA) cycle. Accelerated glucose uptake and consumption, in parallel with inhibition of pyruvate oxidation and its conversion into lactate in the presence of O_2_, guarantee the continuous ATP production to satisfy energetic requirements of highly proliferative cells and contextually supply some intermediates for biosynthesis of macromolecules, such as lipids, nucleotides, and amino acids, preserving redox homeostasis as well [9]. Nevertheless, like glycolytic intermediates, TCA intermediates are also used as precursors for macromolecule synthesis, but their availability is not enough under increased glycolytic flux conditions. Thus, tumor cells activate anaplerotic pathways, such as glutaminolysis and pyruvate carboxylation, for “refilling” the TCA cycle with its lacking intermediates [10].

**Figure 1:**
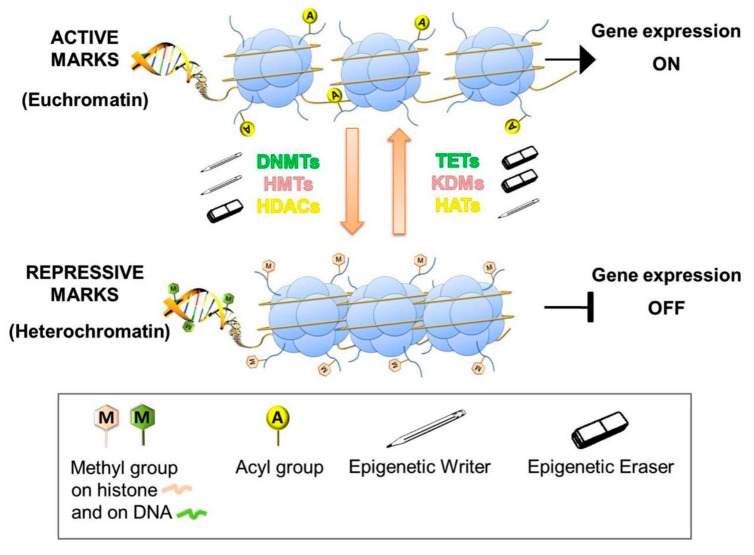
Most representative epigenetic chromatin modifications. The most representative epigenetic modifications on chromatin are histone acetylation and DNA/histone methylation. Epigenetic enzymes (writers or erasers) introduce or remove chemical tags, reversing chromatin shape from euchromatin to heterochromatin or vice versa, thus causing changes in gene expression. DNA methyltransferases [DNMTs] and Ten Eleven Translocation hydroxylases [TETs], respectively, add or remove the methyl groups on DNA. Histone methyltransferases [HMTs] and lysine demethylases [KDMs] are responsible for histone methylation/demethylation, whereas histone acetyltransferases [HATs] and deacetylases [HDACs] are the competitors for, respectively, addition or removal of acetyl groups at histone lysine residues.

Several intrinsic and extrinsic signals can shape the plasticity of cancer cells driving transformation and progression by reprogramming or rewiring cellular metabolism [11]. Among numerous intrinsic factors (i.e., genetic alterations, cell/tissue of origin, tumor grade, histological subtype), which impact potentially on cancer metabolic alterations, in the last years, the interplay between cellular metabolism and gene expression regulation has fascinated the scientific community because their strict coordination allows cancer cells to comply rapidly with ever-changing environments [12]. Indeed, epigenetic regulation of gene expression is one of the most highly and quickly responsive mechanisms available for cell dynamic adaptation to external stimuli and environmental changes. Epigenetic effectors, known as *writers*, *readers,* and *erasers*, shape chromatin through reversible post-translational modifications of histones and DNA, making it easily accessible or closed off to transcription factor complexes [13] (Figure 1). Similar to other enzymes, epigenetic effectors require the availability of cofactors or substrates, generally intermediary metabolites, to perform their remodeling activity; additionally, several metabolic intermediates can modulate their tasks, inhibiting them or affecting their specificity for the substrate [14,15,16,17]. Undoubtedly, the tight regulation of epigenetic processes for the correct development of organisms, as well as their crucial role in changing the epigenome of cancer cells, has been extensively demonstrated [18,19,20]. Aberrant epigenetic patterns are crucial in tumorigenesis, especially DNA and histone methylation and histone acetylation, for their potential role in silencing tumor suppressor genes and/or activating oncogenes. Consequently, the effect of metabolic alterations in cancer cells, changing availability of intermediates with a part in epigenetic modulations, might affect cellular epigenome in an uncontrolled manner, thus producing a cascade of unpredictable effects [14,15,16,17]. Also, many pieces of evidence suggest that the interplay between epigenetic mechanisms and metabolic changes is bidirectional [2,4,21,22]. Indeed, as tumor metabolism provides molecules able to control and influence epigenetic mechanisms and sustain tumor progression, equally epigenetic modifications might regulate the metabolism of cancer cells, inducing a metabolic reprogramming to achieve rapidly the best responses to adverse environmental conditions [22]. In such a view, this bidirectional communication between metabolism and epigenetics is still a matter of research due to its complexity and numerous implications in cellular physiology. This review aimed to describe how the bidirectional interaction between cancer metabolism and epigenome might promote cancer initiation and progression. A better comprehension of their underlying roles in the acquisition of malignant phenotypes might help to identify new potential molecular strategies to target this crosstalk and counteract tumor progression.

## 2. As the Metabolic Rewiring Controls the Epigenome

**Figure 2:**
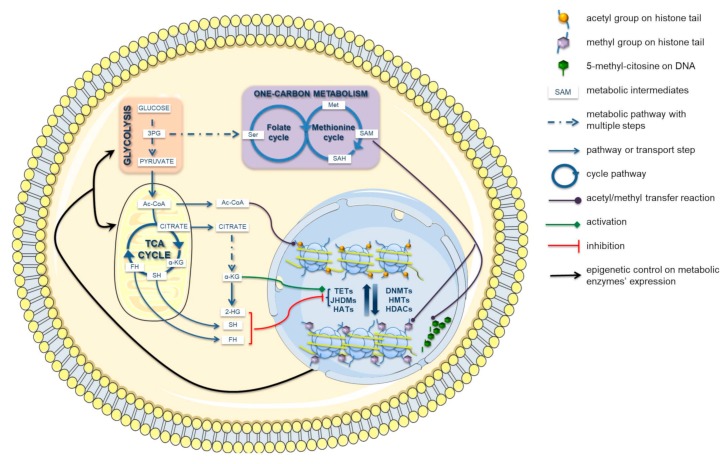
Crosstalk between metabolism and epigenetics. Numerous metabolic intermediates contribute to epigenetic machinery as cofactors, like α-ketoglutarate [α-KG] for Ten Eleven Translocation hydroxylases [TETs] and Jumonji C domain-containing histone demethylases [JHDMs] activity, or as substrates, such as S-adenosyl-methionine [SAM] in DNA and histone methylation and acetyl-CoA [Ac-CoA] in histone acetylation. Moreover, metabolic reprogramming in cancer cells might promote the accumulation of particular metabolites, such as 2-hydroxyglutarate [2-HG], fumarate [FH], and succinate [SH], with tumorigenic driving effect due to their ability to interfere with epigenetic effectors. Nevertheless, epigenome alterations may influence cellular metabolism, controlling the expression of key metabolic enzymes involved in cancer metabolic reprogramming. DNMTs: DNA methyltransferases; HMTs: Histone methyltransferases; HATs: Histone acetyltransferases; HDACs: Histone deacetylases; TCA: Tricarboxylic acid; SAH: S-adenosyl homocysteine; PG: phosphoglycerate.

The metabolic reprogramming of tumor cells can affect the epigenetic landscape (Figure 2) through, at least, three major mechanisms: (1) altering the cellular concentration of specific metabolites, which act as epigenetic cofactors or substrates; (2) generating alternative metabolites, known as oncometabolites, whose accumulation drives cancer growth and progression due to their ability to inhibit or activate epigenetic enzymes; (3) producing epigenetic cofactors or applying epigenetic modifications directly into nucleus by means of translocated metabolic enzymes [23,24].

### 2.1. Metabolites as Cofactors and/or Substrates of Epigenetic Players

The epigenetics control gene expression, through hundreds of distinct covalent modifications on DNA and histones, is carried out by different enzymes. The addition or removal of these marks is strictly managed by epigenetic players through the availability of substrates, cofactors, or allosteric regulators. Aside the severe effects of metabolic rewiring in cancers, the resulting alteration of physiological levels of specific metabolites have a notable impact on the epigenetic machinery because it affects enzymatic parameters, such as K_m_, V_max_, and allosteric and inhibitory binding constants, thus altering enzyme activities or kinetic and thermodynamic properties of epigenetic reactions [15].

The most common metabolic intermediates of epigenetic methylation and acetylation processes are S-adenosyl methionine (SAM), acetyl-coenzyme A (acetyl-CoA), nicotinamide adenine dinucleotide (NAD^+^), α-ketoglutarate (α-KG), and flavin adenine dinucleotide (FAD). These metabolites are strictly connected with glycolysis and TCA cycles, the major metabolic pathways affected by cancer metabolic reprogramming; thus, they could be critical players in the crosstalk between metabolism and chromatin remodeling (Figure 2).

DNA and histones methylation requires the universal methyl donor SAM, produced in the methionine cycle that forms the ‘one-carbon metabolism’ with the folate cycle. After the transfer of a methyl group, SAM is converted to SAH (S-adenosyl homocysteine), which is recycled in the methionine cycle via hydrolysis in homocysteine. The carbon units that feed the one-carbon metabolism derive from specific amino acids, i.e., threonine, serine, glycine, and methionine. In particular, serine metabolism is frequently dysregulated in cancer [25,26] and gives a significant contribution to tumor homeostasis [27], growth, and proliferation [25]. Many pieces of evidence support interconnection between glycolysis and serine synthesis: 3-phosphoglycerate (3PG), an intermediate metabolite of glycolysis, can be shunted into serine metabolism for amino acid biosynthesis, thus, fueling cells of precursors for SAM synthesis [28]. Methylation status of histones and DNA is sensitive to the SAM/SAH ratio because SAM is an essential co-substrate for methyltransferase activity of DNA methyltransferases (DNMTs) and histone methyltransferases (HMTs), while SAH is a potent inhibitor of all methyltransferases. Alterations in the SAM/SAH ratio impact profoundly on chromatin methylation, producing aberrant expression profiling. Indeed, increased SAM level is linked with DNA hypermethylation at CpG sites and gene silencing of several key genes implicated in cancer progression and metastasis [29,30,31,32]. In conditions of methionine restriction, the intracellular SAM concentration is low, and the global methylation of several histone lysines is decreased, in particular, H3K4me3, which is associated with open chromatin and active transcription. In cancer cells, the effect of methionine starvation is negligible on genome-wide distributions of H3K4me3 peaks, but it is considered on the height and area of H3K4me3 peaks, which is correlated with modulation of genes involved in cell cycle progression and cancer-related pathways [33].

Histone acetylation is a dynamic and reversible modification regulated by the competing activity of histone acetyltransferases (HATs) and deacetylases (HDACs) that, respectively, add or remove acetyl groups at histone lysine residues. The activity of these enzymes is sensitive to the availability of metabolites that act as substrates or allosteric regulators. Acetyl-CoA is the acetyl group donor for acetylation of histones. It is an important metabolite synthetized in different subcellular compartments (mitochondria, cytosol, and nucleus) from several sources: pyruvate, acetate, fatty acid β-oxidation, amino acid catabolism. The intracellular concentration of acetyl-CoA is dynamic and strictly dependent on nutrient availability and cellular energy status. The changes in acetyl-CoA abundance can affect histone acetylation levels and gene expression [12,16], especially in cancer cells [34,35]. Indeed, acetyl-CoA modulates kinetic and binding parameters of epigenetic writers and erasers involved in histone acetylation. HATs have a similar affinity for their cofactor, acetyl-CoA, and their inhibitor, CoA, a product of histone acetylation reaction. This indicates that the acetyl-CoA/CoA ratio is the crucial regulator of the enzymatic activity and specificity of HATs, rather than the absolute levels of acetyl-CoA [36]. Many HATs have also a relatively high dissociation constant K_D_ (low affinity) for acetyl-CoA, thus the physiological fluctuations in the abundance of acetyl-CoA within the cellular compartment, where such acetyltransferases are expressed, can affect their catalytic activity and histone acetylation levels [36,37].

The oncogene-driven metabolic reprogramming of cancer cells stimulates high glycolytic flux furnishing cells of pyruvate, essential for acetyl-CoA synthesis. It has been demonstrated that the abundance of acetyl-CoA triggers epigenetically the up-regulation of a gene involved in cell cycle progression, proliferation, cell migration, metabolism, and macromolecular biosynthesis, upon remodeling of chromatin structure [16,34,35]. Additionally, the expression of acetyl-CoA synthase enzymes (i.e., ATP citrate-lyase and acetyl-CoA synthase short-chain family member 1) is frequently upregulated in cancer cells, leading to elevated intracellular acetyl-CoA levels and, in turn, histone acetylation, an epigenetic mark associated with open chromatin and active gene expression [12]. Moreover, a positive feedback control loop of acetyl-CoA derived from glucose on genes connected with glucose uptake and metabolism has been observed, as well as acetyl-CoA from acetate on genes involved in lipid biosynthesis [16].

Furthermore, in their metabolic rewiring, cancer cells, unable to perform normal oxidative phosphorylation (OXPHOS) in mitochondria, use the glutamine reductive carboxylation to provide both energy and carbon source for cancer growth and replenish the TCA intermediates for macromolecules biosynthesis. This catabolic pathway also produces citrate and acetyl-CoA and represents the dominant metabolic pathway for cytosolic acetyl-CoA, refueling in rapidly growing malignant cells, with mutation or inhibition of electron transport chain [38].

The metabolic rewiring in cancer cells might also control histone deacetylation to promote gene silencing. There is some evidence that histone deacetylation is influenced by the intracellular pH (pH_i_). Under low pH_i_, histone deacetylation catalyzed by HDACs is accelerated, and released acetate anions are co-exported with protons in the extracellular environment to maintain the intracellular pH and prevent acidification of intracellular space [39], which could inhibit glycolysis by direct and indirect inhibition of phosphofructokinase (PFK) activity [40]. Intriguingly, an alkaline pH_i_, essential for driving aerobic glycolysis, reduces HDACs catalysis, leading to a global histone hyperacetylation.

Another connection between the energetic cells state and histone acetylation is provided by sirtuins, a class of HDACs (class III HDAC), dependent from NAD^+^ levels for their deacetylation activity. The NAD^+^/NADH ratio is closely associated with the energy status in cells. Indeed, when energy is plentiful, like in cancer cells where the glycolytic activity is intensive, the NAD^+^/NADH ratio drops down, inhibiting the sirtuin catalysis [12]. The low NAD^+^/NADH ratio, together with an increase of HATs activity for high acetyl-CoA levels, may contribute to histone hyperacetylation and aberrant gene expression in tumors.

Additionally, the activity of Zn^2+^-dependent class I, II, and IV HDACs could be influenced by metabolism due to sensitivity to the inhibitory effect of butyrate and its derivatives [41], produced by gut microbiota fermentation in the colon. Generally, butyrate is metabolized by β-oxidation, followed by TCA cycle in colonocytes. Nevertheless, cancerous cells are unable to metabolize butyrate efficiently; thus, this metabolite is accumulated in the nucleus where it inhibits HDACs, upregulating the expression of downstream target genes. The effect of HDACs butyrate-dependent inhibition on cell growth depends on cell metabolism [42]. It inhibits cell proliferation as an HDAC inhibitor in cancer, but stimulates the proliferation of noncancerous cells, being a source of acetyl-CoA for HATs activity. The critical factor of the “butyrate paradox” is the Warburg effect [42].

α-ketoglutarate is a TCA cycle metabolite involved in many cellular processes due to its function as an obligatory cofactor of 2-oxoglutarate-dependent dioxygenases (2-OGDO), a family of non-heme oxidizing enzymes that catalyze hydroxylation and demethylation of proteins and nucleic acid [43]. Ten-eleven translocation hydroxylases (TETs), involved in DNA demethylation, and the Jumonji C domain-containing lysine demethylases (JmjC-KDMs or JHDM) are the major histone demethylases and are part of the 2-OGDO family, reflecting a strong dependence of DNA and histone methylation on α-KG availability. α-KG is produced from isocitrate in mitochondria, by mitochondrial isocitrate dehydrogenase isoforms IDH2 and IDH3, and in the cytosol, by peroxisomal and cytoplasmatic isoform IDH1, starting from citrate exported by mitochondrial citrate/isocitrate carrier. Alternative sources of α-KG are amino acids, especially glutamine via transamination of derived glutamate. Glucose and glutamine catabolism maintain high levels of α-KG, thus promoting demethylation of H3K27me3, H3K9me3, H4K20me3, and TET-dependent DNA demethylation. This modification on methylation pattern contributes to the regulation of pluripotency-associated genes and supports stem cell self-renewal and pluripotency maintenance [44], even if the effect of α-KG on cells differentiation is strictly dependent on the specific pluripotency stage of stem cells and the physiological context [45]. In a different type of solid cancers (glioblastoma, chondrosarcoma, and cholangiocarcinoma), the decrease of α-KG leads to a dramatic DNA and histone hypermethylation, which is associated to cell dedifferentiation and drug resistance [46,47]. It is noteworthy that the epigenetic mark H3K4me3, regulated by the Trithorax complex [48], is less sensitive to changes in α-KG intracellular concentrations [49] respect to H3K27me2/3 and H3K9me3, mediated by polycomb-group complex 2 (PRC2) [48]. This means that metabolic rewiring of cancer cells could regulate gene expression, targeting a specific subset of genes to sustain tumorigenesis.

The flavin-adenine dinucleotide (FAD)-dependent lysine demethylases (LSD1 and LSD2) remove methyl groups from H3, using as a cofactor FAD, a metabolite derived from riboflavin and ATP [12]. Besides FAD biosynthesis, as a limiting factor for cellular FAD availability, the redox status may also affect cellular FAD levels, and consequently LSD1/2 activity [50]. Indeed, some oxidoreductase enzymes known as flavoproteins (i.e., succinate dehydrogenase, acyl-CoA dehydrogenase, α-ketoglutarate dehydrogenase) reduce FAD to FADH_2_ to oxidize their substrates; thus, they compete with LSD-demethylases for the cofactor FAD reducing their activity. Additionally, during the early phase of hypoxia, an increase of FAD level is observed due to the effect of O_2_ depletion on cellular respiration, whereas, during prolonged hypoxia, FAD drops down because of a negative feedback mechanism of LSD1 inactivity on FAD biosynthetic enzymes [51]. The role of LSD demethylases on cancerogenesis is controversial. Some studies demonstrated that increase in FAD-precursor riboflavin reduces cancer risk in colorectal and cervical cancers. However, promoting LSD1 activity could increase cancer risk since it is essential for the maintenance of the pluripotency in embryonic stem cells [52,53]. The function of LSD2 is less known, but its involvement as a suppressor in lipid metabolism has been suggested [50].

### 2.2. Oncometabolites

In the context of metabolic alterations, malignant cells can change their metabolic status by two mechanisms. One is the “reprogramming” of conventional metabolic pathways, namely enhancing or suppressing their activities respect to normal tissues. The other one is the “rewiring” of the metabolic status, which means the acquisition of metabolic features, not founded in normal cells, ascribable to mutation events [2]. For example, mutations in metabolic enzymes, as fumarate hydratase (FH), succinate dehydrogenase (SDH), and isocitrate dehydrogenase (IDH), cause the production/accumulation of particular intermediary metabolites with oncogenic intracellular signaling function. “Oncometabolite” is a new term coined to indicate these particular metabolites, whose abundance increases markedly in tumors, often caused by loss-of-function or gain-of-function mutations of genes encoding for enzymes involved in their production [54]. Succinate, fumarate, 2-hydroxyglutarate (both enantiomers D and L), and β-hydroxybutyrate are considered oncometabolites due to their driving role on cancer transformation through a profound impact on epigenetic effectors’ activity [55].

β-hydroxybutyrate is a ketone-body with HADCs inhibitory capability, which induces histone H3K9 and H3K14 hyperacetylation and specific changes in gene expression [41]. Its oncogenic role is controversial because its anti-proliferative [56,57] or growth-promoting effect [58,59] depend on the metabolic state of cancer cells [60]. 

The structural similarity of L/D-2-hydroxyglutarate, succinate, and fumarate compared to α-KG makes these oncometabolites antagonist competitors of α-KG for 2-OGDO. Due to their inhibitory effect, these oncometabolites can drive cellular transformation and oncogenesis. As potent inhibitors of TETs and JHDMs, their accumulation causes an epigenetic dysregulation due to DNA and histone hypermethylation and triggers a transcriptional program with downregulation of genes involved in suppression of metastasis and simultaneously promotes dedifferentiation, epithelial-mesenchymal transition (EMT), and invasiveness [55,61,62,63]. Loss-of-function mutations of SDH and FH enzymes cause the accumulation of, respectively, succinate and fumarate in different types of human malignancies. Instead, gain-of-function mutations of IDH1 and IDH2 isoforms lead to the production and accumulation of L- and D-2-hydroxyglutarate (L-/D-2HG) in acute myeloid leukemia, glioma, chondrosarcoma, and cholangiocarcinoma. An unexpected role in the accumulation of the oncometabolite succinate is played by the molecular chaperone tumor necrosis factor receptor associated protein 1 (TRAP1), which inhibits SDH, thus contributing to the downregulation of mitochondrial respiration [64,65,66]. Marked up levels of succinate induce widespread alterations of epigenetic landscape due to TETs and JHDMs inhibition. Fumarate accumulation, caused also by reduced expression of FH in cancer [67], interferes with TET-mediated DNA demethylation in a regulatory region of an anti-metastatic miRNA (mir-200ba429), leading to the expression of EMT-related transcription factors and promoting migration [68]. Also, the tumor microenvironment contributes to metabolic rewiring in wild-type IDH1/2 tumors, that under hypoxia generate the oncometabolite L-2-HG through a ‘promiscuous’ reduction of α-KG [69]. The acid intracellular environment, a common feature of cells with active glycolysis, stimulates lactate dehydrogenase A (LDHA) and, in a lesser manner, malate dehydrogenase (MDH), to produce L-2-HG using the alternative substrate α-KG. When pH is decreased, the K_m_ of LDHA for α-KG is reduced by about four-fold [70], hence the enzyme’s affinity for this substrate increases.

Therefore, it can be concluded that the accumulation of oncometabolites is necessary and sufficient for alterations of the methylation status of both DNA and histones, as well as for histone acetylation, leading to epigenetic modifications in gene expression.

### 2.3. Metabolic Enzymes Moonlighting in the Nucleus

Nowadays, a new paradigm of gene regulation is emerging in which the nuclear localization of specific metabolites or metabolic enzymes can modulate epigenetics to target the expression of nearby genes [71,72]. The subcellular localization of metabolic effectors and/or products might supply the essential intermediates to epigenetic machinery locally to direct epigenetic modifications in specific chromatin regions. For example, the nuclear production of the methyl donor SAM, generally, biosynthesized in the cytoplasm, has been observed in cancer cells. Splicing variants of MATs (S-adenosylmethionine synthetase) with nuclear localization have been found [73], and redox stress might be a putative mechanism to control the subcellular localization of these enzymes [74]. The hypothesis is that the nuclear translocation of MATs provides SAM production locally to support epigenetic methyltransferases activity and direct epigenetic methylation processes at target sites. The physical association of MATIIα with some chromatin- and transcription-related factors, forming a complex known as serine-responsive SAM-containing metabolic enzyme complex [SESAME] [75,76,77], reinforces the above hypothesis that local generation of methyl donors can have relevant functions for targeted epigenetic repression.

Acetyl-CoA is compartmentalized into mitochondria and cytosol, but it can diffuse freely through the nuclear pores; thus, the nuclear synthesis would not be needed. However, under particular environmental or cellular conditions [78,79], four enzymes involved in acetyl-CoA synthesis, including ACSS2 (Acetyl-CoA synthetase short-chain family member 2) [80,81], ACLY (ATP citrate-lyase) [34,82], PDC (pyruvate dehydrogenase complex) [83,84], and CAT (carnitine acetyltransferase) [85], can transiently localize to the nucleus, thus increasing the local concentration of acetyl-CoA, even if its total intracellular level does not change significantly. The nuclear translocation of these enzymes has been detected in different cancer cell lines with an impact on transcriptional programs in a context-dependent manner (i.e. nutrition, disease, microenvironment) [82].

Numerous glycolytic enzymes can moonlight in the nucleus where they perform autonomously and often play unrelated functions involved in epigenetic regulation of gene expression [23]. The pyruvate kinase embryonic isozyme M2 (PKM2), resulting from alternative splicing of PKM pre-mRNA, is a promoter of Warburg effect [86] and it is over-expressed in different types of cancer [87]. The tumor microenvironment can promote cell proliferation and reprogramming of cancer metabolism, controlling epigenetic processes as well [86,88,89] and providing suitable stimuli, as growth stimuli [88] or hypoxia [90], for PKM2 nuclear translocation. Here, it works synergistically with nuclear pyruvate dehydrogenase complex (PDC), sustaining histone acetyltransferases activity by locally acetyl-CoA production [91]. PKM2 might also modulate histone methylation, as demonstrated by Li and colleagues who found Pyk1, the yeast PKM2 homolog, to participate in the upregulation of H3K4me3 as member of the SESAME complex [77].

The metabolic enzyme 6-phosphofructo-2-kinase/fructose-2,6-bisphosphatase 4 (PFKFB4), highly expressed in cancer cell lines under hypoxia [92], regulates cellular levels of fructose-2,6-bisphosphate (F2,6BP), an important sugar-phosphate metabolite that stimulates glycolysis by allosteric activation of PFK1. PFKFB4 contributes to cell survival and tumor growth [93], increasing the amount of F2,6BP and ATP, whose cellular production is limited by electron transport chain blockage. Recently, Dasgupta and colleagues reported a moonlight kinase function of PFKFB4, activated by the Warburg effect, on steroid receptor coactivator-3 (SRC-3) [94], a transcriptional co-activator with several nuclear receptor interacting domains and intrinsic histone-acetyltransferase activity. PFKFB-phosphorylated SRC-3 promotes the expression of genes involved in driving glucose flux towards purine synthesis, a critical determinant of metastatic progression in breast cancer [94].

Mitochondrial and nuclear membranes are impermeable to NAD^+^ and NADH, so the regulation of NAD^+^/NADH ration, a critical factor for various epigenetic processes, is compartmentalized. Nuclear NAD^+^ availability may be guaranteed by the metabolic enzymes LDH and glyceraldehyde 3-phosphate dehydrogenase (GAPDH), whose compartmentalization in the nucleus has been demonstrated. It has been established that the nuclear translocation of LDHA, induced by ROS stress, is crucial to activating gene transcription by histone deacetylation, modulating the activity of sirtuin 1 (SIRT1) through the availability of NAD^+^ directly in the nucleus [95]. Moreover, GAPDH can activate a particular apoptotic pathway promoting histone acetylation under intracellular or external stress stimuli [96]. Nitric oxide promotes GAPDH S-nitrosylation, which induces its binding with the protein Siah1 and its nuclear translocation [97]. In the nucleus, S-nitrosylated GAPDH, in turn, transnitrosylates both HDAC1, promoting its dissociation from chromatin and gene transcription activation, and SIRT1, inhibiting its activity and, consequently, the autophagy program [98].

Unlike the glycolytic enzymes, only few TCA enzymes translocate from mitochondria to the nucleus and take part in epigenetic rewiring. Nuclear translocation of mitochondrial proteins could represent a simple and direct mechanism of retrograde communication between the two organelles, which also involves epigenetic processes. Fumarate hydratase is a TCA cycle enzyme with a nuclear ‘echoform’ that regulates the response to DNA damage [99], inhibiting lysine demethylase 2B (KDM2B) histone demethylase activity through local fumarate production [100]. The nuclear accumulation of fumarate inhibits the activity of other epigenetic erasers, influencing the epigenetic landscape and contributing to tumorigenesis. IDH3, a mitochondrial heterotetrameric enzyme, is a rate-limiting step of the TCA cycle. Despite its central role in TCA cycle, recently a group of researchers found the accumulation of IDH3α in the cytosol and nuclear periphery in S phase-arrested cells. Here, IDH3α colocalizes and interacts with serine hydroxymethyltransferase (SHMT) and, in this way, it regulates locally the one-carbon metabolism, modulating pyrimidine synthesis as well as DNA methylation. Loss of IDH3α function results in an increase of the methyl group donor SAM and DNA methylation level due to a decrease of α-KG availability for TET activity [101]. The cytoplasmatic isoform IDH1 is found in the nuclei of glioma cells [102], likely involved in histone and DNA methylation. At present, the specific role of nuclear IDH1 is still unclear, but its contribution to producing NADPH from NADP^+^ is worthy of attention, whose ratio may modulate gene expression by initiating redox signaling [103].

## 3. As Epigenetics Control Metabolic Reprogramming

Epigenetic dysfunction is another rising hallmark of malignancy, even though its effect on human carcinogenesis is not entirely acknowledged. Indeed, besides genetic mutations, epigenetic alterations are the cause of metabolic enzymes deregulation in cancer cells since epigenetic modulation of metabolic enzymes represents an efficient mechanism to obtain a reversibly and rapid response to environmental short-term changes. Epigenetics can regulate cellular metabolism directly, controlling transcription of metabolic genes, or indirectly, dysregulating oncogenic cascades, as AKT serine/threonine kinase (AKT), AMP-activated protein kinase (AMPK), or hypoxia-inducible factor 1 (HIF1) signaling.

**Figure 3:**
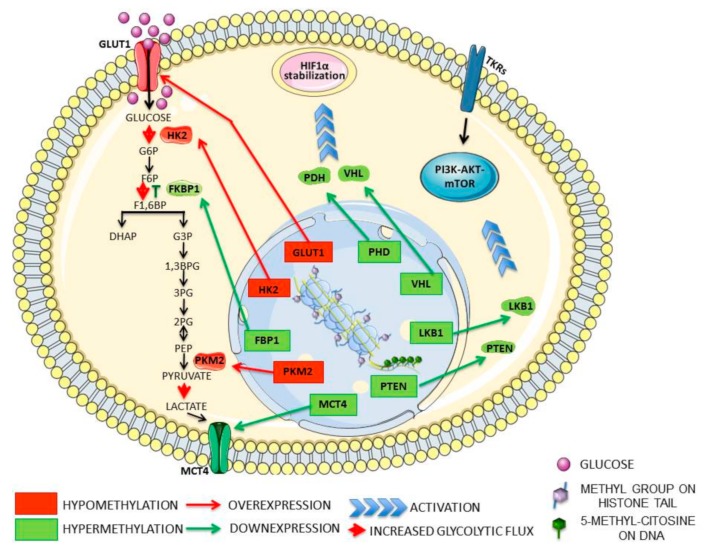
Direct and indirect epigenetic control of cellular metabolism. The DNA and histones methylation status influences the glycolytic flux through promoter hypermethylation or hypomethylation of enzymes with key-roles in the glycolytic pathway. Indeed, hypomethylation of PKM2 (pyruvate kinase embryonic isozyme M2) or HK2 (hexokinase 2) promoters is associated with the high glycolytic flow, whereas the downregulation of fructose 1,6-biphosphatase (FBP1) expression, due to promoter hypermethylation, inhibits gluconeogenesis and favors glycolytic metabolism. Moreover, epigenetic mechanisms indirectly control cell metabolism by promoter hypermethylation and consequent silencing of numerous tumor suppressor genes that repress PI3K/AKT/mTOR and HIF1α signaling pathways, two pro-tumorigenic cascades involved in prompting Warburg metabolism. Additionally, numerous histone demethylases, remodeling histone methylation patterns, influence cellular metabolism promoting transcriptional activation of several glycolytic genes, such as glucose transporter type 1 (GLUT1) and monocarboxylate transporter 4 (MCT4), as well as mediating the activity and/or availability of crucial transcription factors. Abbreviations: G6P-Glucose 6-Phosphate; F6P-Fructose 6-Phosphate; F1,6BP - Fructose 1,6-Biphosphate; DHAP Dihydroxyacetone Phosphate; G3P-Glyceraldehyde 3-Phosphate; 1,3BPG-1,3-Biphosphoglycerate; 3PG-3-Phosphoglycerate; 2PG-2-Phosphoglycerate; PEP-Phosphoenolpyruvate; TKRs-Tyrosine Kinase Receptors; PTEN-phosphatase and Tensin homolog; LKB1- Liver kinase B1; VHL-Von Hippel-Lindau tumor suppressor; PHD-Prolyl Hydroxylase Domain-containing protein.

Numerous evidence link DNA methylation status and glycolysis. For example, the upregulation of the glycolytic enzyme PKM2 is associated with hypomethylation of its promoter 1 in several cancer types [87]. Promoter hypomethylation of hexokinase 2 (HK2), a Warburg effect mediator, increase HK2 gene transcription and protein availability, favoring glycolytic flux in hepatocellular carcinoma [104,105] and glioblastoma [106,107]. Furthermore, DNA hypermethylation mediates silencing of fructose 1,6-biphosphatase (FBP1), thus inhibiting gluconeogenesis and inducing higher glycolytic rates in gastric, colon, and liver cancers (Figure 3) [108]. DNMTs are recruited at FBP1 promoter by transcription factors cooperating with histone writers and erasers, such as HMTs (G9a and SUV39H1) [109,110], or HDACs (LSD1) [111]. In glioblastoma, the promoter regions of other glycolytic genes, including *ENO1*, *GAPDH*, *HKI-III*, and *LDHA*, have been found hypermethylated in IDH1-mutant cells [107]. Epigenetics may modulate indirectly cancer metabolic reprogramming via overexpression of the glucose transporter, GLUT1, due to promoter hypermethylation of derlin-3, a degradation mediator of the solute carrier [112]. Epigenetic silencing of derlin-3 promotes Warburg effect and tumorigenesis.

DNA methylation contributes indirectly to the Warburg effect, driving transcriptional silencing of tumor suppressor genes involved in signaling cascades linked to tumor metabolism. PI3K/AKT/mTOR and HIF1 signaling play crucial roles in the activation of glycolysis and other cancer-related metabolic pathways, acting as bioenergetic sensors. Several tumor suppressors that repress PI3K/AKT/mTOR and HIF1 signaling are epigenetically silenced by promoter hypermethylation, including *PTEN* [113,114,115,116,117], *LKB1* [118,119], *VHL* [120,121,122], and *PHD*1/2/3 [123,124] (Figure 3). Hence, PI3K/AKT/mTOR pathway and HIF1-mediated hypoxic response are constitutively activated, contributing to glycolytic phenotype in cancer cells.

Several histone demethylases take an active part in metabolic rewiring in human cancers, as well. Lysine demethylases (KDMs) are often overexpressed and constitutively activated in solid tumors. For example, in bladder cancer, KDM3A overexpression is associated with the metabolic shift to glycolysis because the enzyme catalyzes H3K9me2 demethylation of glycolytic genes’ promoters, including *SLC2A1*, *HKII*, *PGK1* (phosphoglycerate kinase 1), *LDHA,* and *SL16A4* (monocarboxylate transporter 4), leading to their transcriptional activation [125]. KDM4C overexpression is associated with increased glycolytic metabolism through HIF1α interaction in breast cancer [126]. Active LSD1 is implicated in the inhibition of gluconeogenesis through H3K4me2 demethylation, leading to FBP1 and G6P transcriptional repression [111]. Moreover, LSD1, decreasing the methylation of H3K4 at the v-Myc avian myelocytomatosis viral oncogene homolog (MYC) locus, elevates its expression, leading to glycolytic shift [127]. Among the enzymes involved in histone modification, the role of sirtuins in cell metabolism reprogramming has been extensively studied. SIRT6 controls glucose homeostasis by modulating histone acetylation [128]. SIRT6 deletion is frequent in several human cancers, like colon, pancreatic, and hepatocellular carcinomas, and it is associated with the increase of H3K9 acetylation and upregulation of glycolytic genes [128,129]. Additionally, SIRT6 interacts directly with HIF1α and MYC, repressing HIF1-mediated glycolytic switch and MYC-dependent ribosome biogenesis and glutaminolysis [21]. SIRT1 is another sirtuin with tumor-suppressor function. It represses glycolytic metabolism, indirectly through HIF1α deacetylation and directly by inhibiting the glycolytic enzyme PGAM1 (phosphoglycerate mutase 1) via deacetylation [130]. SIRT2 takes part in metabolic dysregulation in cancer, indirectly stabilizing MYC [131]. Upon deacetylation of histone H4K16, SIRT2 suppresses the transcription of neural precursor cell expressed developmentally down-regulated 4 (NEDD4), a negative regulator of N-MYC and C-MYC, promoting their ubiquitination and proteasomal degradation [131].

Epigenetics might regulate indirectly OXPHOS in cancer, impairing mitochondrial functions as the last result. Even if researches in this field are still at an early stage, some evidence is heading the interest toward this issue. For example, the histone methyltransferase set domain containing lysine methyltransferase 7 (SETD7) or histone demethylases LSD1 and KDM5 are epigenetic enzymes whose activity or inactivity regulates mitochondrial function and/or gene expression [132].

The direct and indirect mechanisms implemented by epigenetics for metabolism control are numerous and not completely identified, so much remains to be understood concerning the role of epigenetic factors in prompting cancer metabolic rewiring and/or reprogramming.

## 4. Metabolic/Epigenetic Changes Modify Tumor Microenvironments Promoting Immune Escape and Tumor Progression

The metabolism-epigenetics interplay needs to be discussed also in the dynamic context of the interactions between cancer cells and tumor microenvironment (TME) because they influence each other. Metabolic and epigenetic changes, occurring in cancer cells, contribute to shape tumor microenvironment (TME) and surrounding cell phenotype (i.e., fibroblasts and myofibroblasts, neuroendocrine, adipose, immune, endothelial and inflammatory cells, blood and lymphatic vascular networks, and extracellular matrix), eliciting immune tolerance, drug resistance, and, consequently, tumor progression [133,134]. The imbalanced metabolism of cancer cells results in excessive production and secretion of various metabolites, among which the most important is lactic acid [134,135]. High lactate levels in TME, together with low oxygen and nutrients, seems to play an important role in enhancing the immunosuppressive activity of macrophages, T cells, myeloid-derived suppressor cells (MDSCs), and other immune cells [134,136,137]. Recent studies showed that hypoxia and high levels of lactate in TME influence the phenotype of tumor-associated macrophages (TAMs) and, in particular, their differentiation and polarization towards the M2 phenotype [138]. Indeed, TAMs-M2 express low levels of major histocompatibility complex class-II (MHC-II), high levels of Arginase-1 (Arg-1), mannose receptor C type 1 (CD206), and vascular-endothelial growth factor (VEGF), thus promoting angiogenesis and tissue remodeling [136,139,140]. Furthermore, in response to high concentrations of lactic acid, TAMs enhance the production of inflammatory cytokines, such as interleukin-10 IL-10, which play an immunosuppressive function [135]. In agreement with these observations, it has been recently suggested that the suppression of lactate dehydrogenase (LDHA) and consequently of lactate in myeloid cells leads to regression of lung cancer and favors a more pronounced anticancer immune response [141]. Consistently, lactate promotes the expression of programmed death-ligand 1 (PDL1) by a mechanism that likely engages the lactate-mediated HIF1α stabilization [141].

The activation of T cells against tumor cells involves their expansion and proliferation, but this process is highly dependent on the availability of oxygen and nutrients whose concentration is extremely limited in TME [142,143]. In particular, hypoxia, low availability of nutrients, and high levels of lactate inhibit T cells expansion and change their metabolism towards glycolysis with consequent loss of their anti-cancer response [143].

Epigenetic changes, occurring in tumor cells, lead to production and secretion of a large variety of cytokines, chemokines, and growth factors, such as prostaglandin E2 (PGE2), IL-6, IL-10, granulocyte-macrophage colony-stimulating factor (GM-CSF), VEGF, and transforming growth factor β (TGFβ), that induce the accumulation of immune and stromal cells with immunosuppressive functions in TME [144,145]. Thus, tumor cells shape TME, driving immunological escape through impairment of the functionality of cytotoxic T lymphocytes (CTLs), Natural Killers (NKs), antigen-presenting cells (APCs), and Dendritic Cells (DCs) [145]. Many proinflammatory cytokines, including interleukins, are epigenetically regulated in human malignancies. In particular, *IL-1B*, *IL6,* and *IL8* genes are downregulated upon promoter methylation in Non-Small-Cell Lung Cancer (NSCLC) [146], whereas IL23, a member of IL6 family, is epigenetically regulated by both histone acetylation and DNA methylation [146]. The epigenetic silencing of these molecules and the consequent lack of their secretion in TME is one of the main mechanisms of tumor immunological evasion, drug resistance, and tumor progression.

Moreover, the metabolic reprogramming of cancer cells, with the consequent remodeling of TME, has an indirect control on the epigenetic machinery of cancer and stromal cells [22,147]. For example, the release of lactate causes the acidification of surrounding microenvironment and negatively regulates HDACs activity and, consequently, gene expression [148]. Instead, glutamine depletion in TME, due to excessive uptake and its metabolization by cancer cells and/or low oxygen levels during hypoxia, inhibits KDMs-mediated histone demethylation processes, leading to repression of histone markers, and consequent downregulation of numerous genes [149].

Thus, besides the effects that metabolic reprogramming or epigenetic alterations have on TME, it is also important to underline the role of TME on the promotion and maintenance of malignant phenotypes. Intriguingly, the metabolic-epigenetics-TME crosstalk seems to work symbiotically favoring tumor proliferation, progression, and drug resistance. This intertwined relationship might offer new perspectives on the role of metabolites, in particular, lactate, and epigenetic regulation of cytokines, chemokines, and growth factors in shaping TME and vice versa. Altogether, these observations suggest a strong rationale for the development of potential therapeutic strategies based on the inhibition of these tumor-promoting mechanisms.

## 5. Epigenetics/Metabolism Crosstalk: New Therapeutic Opportunities

Innovative therapeutic strategies that target the crosstalk between epigenome and metabolome may provide the basis for novel anticancer therapies. To date, most information regarding the effectiveness of metabolic/epigenetic inhibitors is derived from in vitro studies that poorly reflect the complex scenario of epigenome and metabolome interaction in vivo and do not reflect the influence that this crosstalk has on TME. The most interesting cancer therapies, developed recently, have been designed against metabolic targets, with a downstream effect on epigenetics, and against epigenetic effectors with a synergistic effect on cancer metabolic reprogramming.

For example, to reshape aberrant DNA and histone methylation patterns of cancer cells, it is possible to modulate the SAM/SAH ratio acting on SAM synthesis pathway. Interestingly, 3-deazaneplanocin A (DZNep), the inhibitor of s-adenosylhomocysteine hydrolase (AHCY), the enzyme which catalyzes the hydrolysis of SAH into adenosine and homocysteine, participating in the maintenance of methylation homeostasis, causes the reduction of SAM, which is essential for the activity of DNMTs and HMTs [150]. In combination with the DNMTs inhibitor, 5-aza-2′-deoxycytidine (5-Aza), DZNep showed synergistic anti-tumor activity in leukemia, through the reactivation of the expression of genes aberrantly silenced by histone and DNA methylation [151,152]. Instead, to counteract the aberrant DNA hypermethylation caused by 2-HG production in IDH1/2 mutant cancers, promising IDH1/2 mutant inhibitors are available, and Ivosidenib has been recently approved from FDA for acute myeloid leukemia [153]. Among several IDH1/2 mutant inhibitors, AG-120, AG-881, ML309, GSK321, and GSK864 exhibited remarkable anti-tumor activity [21], showing a significant histone and DNA demethylation activity in vitro. Moreover, AG-221 is a specific inhibitor of the IDH2 mutant, currently under phase I and II investigations, which is shown to reduce 2HG levels in plasma and bone marrow and induce durable remissions in patients with IDH2 mutant advanced hematologic malignancies [21,154].

Histone acetylation is dependent on glycolytic flux and glutaminolysis, thus promising therapeutic strategies are based on the modulation of acetyl-CoA levels upon inhibition of these two metabolic pathways. 2-deoxyglucose (2-DG), a glucose analog, suppresses acetyl-CoA levels, leading to a global reduction of histones H3 and H4 acetylation and sensitizing tumor cells to DNA damaging agents [155]. Another glycolytic inhibitor—3-bromopyruvate—reduces acetyl-CoA levels and induces differentiation in embryonic stem cells [156]. Instead, glutaminase (GLS) inhibitors, including bis-2-(5-phenylacetamido-1,2,4- thiadiazol-2-yl) ethyl sulfide (BPTES) [157], CB-839, and compound 968, can alter the acetylation of histones H4 and H3 and downregulate the expression of many tumor-related genes in breast cancer [158]. Additionally, a compound studied as IDH1 inhibitor, Zaprinast, showed an unexpected GLS inhibitory activity [159]. Surprisingly, the molecular mechanism of this drug consists mainly of remodeling histone methylation rather than histone acetylation, probably because GLS-mediated glutaminolysis plays a crucial role in providing α-KG for methyltransferases activity [160].

It has been widely demonstrated that several targeted epigenetic agents, in use for cancer treatment, might affect cancer metabolism as a secondary effect. This is the case of HDAC inhibitors, which have a direct suppressing effect on glucose metabolism. Studies conducted on colorectal, breast, and lung cancer cell lines reveal that the treatment with HDAC inhibitors, such as butyrate and trichostatin A (TSA), causes a reduction in glucose uptake, glycolytic flow, and lactate production and triggers a shift towards oxidative phosphorylation [161]. Instead, use of DNMTs inhibitors, such as 5-Aza, seems to reverse the methylation in IDH-mutant tumors, suppressing tumor growth and inducing differentiation in IDH mutant glioma cells, but their effect on tumor metabolism is not yet known [162,163]. 

Altogether, these data, although preliminary, suggest that metabolic/epigenetic targeting may represent a valid anticancer strategy.

## 6. Conclusions

Despite genetic and tissue-specific heterogeneities, tumor initiation and progression involve common dysfunctions in specific genes and crucial biological functions acquired during a multistep process that drives the transformation of normal into malignant cells. In such a context, the recently defined hallmark of “metabolic rewiring” [164] has established a central role for metabolism as a driver of the malignant phenotype responsible for providing to tumor cells a selective advantage respect to the normal counterpart. Noteworthy, metabolic reprogramming is not a merely passive consequence of cancer transformation finalized to support the core demands of rapidly proliferating cells, as anabolism, catabolism, and redox balance, but it is an active driver of carcinogenesis responsible for favoring rapid adaptation of cancer cells to environmental modifications. Indeed, it contributes to the regulation of genes essential for cell growth, survival, differentiation, and overall cell homeostasis and influences the epigenetic machinery through the regulation of steady-state levels of metabolites with epigenetic involvement. Per contra, epigenetics aberrations, another rising hallmark of cancer [20], play an important role in the etiology of cancer, modulating the expression of oncogenes and/or oncosuppressor genes implicated in cancer initiation and progression. Additionally, epigenetics promote cancer metabolic rewiring through reprogramming of genes with metabolic functions, thus resulting in remodeling of metabolic pathways in the perspective of tumor development.

Therefore, it is incontrovertible that epigenetics and metabolism interact dynamically and reciprocally with the purpose to rapidly shape phenotype of cancer and environmental cells in response to multifactorial intracellular and extracellular stimuli. Deep comprehension of this bidirectional crosstalk could represent an important step towards the design of specific and effective therapeutic strategies to target the metabolism-epigenetic network in malignant cells. In such a perspective, three alternative therapeutic approaches have been proposed:

1. To target epigenetics to remodel cancer cell metabolism and, thus, use epigenetic inhibitors [161,165,166,167] as a surrogate of metabolic agents;
2. To target metabolic pathways to inhibit the production of specific metabolites with epigenetic functions [155,156,158,159,168,169,170,171,172] and, thus, remodel the expression of key cancer-related genes;
3. To simultaneously target epigenetic and metabolic pathways by combination therapies [173,174] to inhibit dynamic adaptive mechanisms of tumor cell reprogramming and obtain synergistic effects.

In conclusion, while there is a strong biological rationale to target the epigenetic-metabolic crosstalk as anticancer strategy and several preclinical studies validated this approach, further studies are needed to design innovative and effective epigenetic and metabolic agents, with acceptable toxicity profile, that can be evaluated in clinical studies.

## References

[1] Cairns R.A., Harris I.S., Mak T.W. (2011). Regulation of cancer cell metabolism. Nat. Rev. Cancer.

[2] DeBerardinis R.J., Chandel N.S. (2016). Fundamentals of cancer metabolism. Sci. Adv..

[3] Cantor J.R., Sabatini D.M. (2012). Cancer cell metabolism: One hallmark, many faces. Cancer Discov..

[4] Martín-Martín N., Carracedo A., Torrano V. (2018). Metabolism and transcription in cancer: Merging two classic tales. Front. Cell Dev. Biol..

[5] Pavlova N.N., Thompson C.B. (2016). Perspective the emerging hallmarks of cancer metabolism. Cell Metab..

[6] Hanahan D., Weinberg R.A. (2011). Hallmarks of cancer: The next generation. Cell.

[7] Matassa D.S., Amoroso M.R., Lu H., Avolio R., Arzeni D., Procaccini C., Faicchia D., Maddalena F., Simeon V., Agliarulo I. (2016). Oxidative metabolism drives inflammation-induced platinum resistance in human ovarian cancer. Cell Death Differ..

[8] Denise C., Paoli P., Calvani M., Taddei M.L., Giannoni E., Kopetz S., Kazmi S.M., Pia M.M., Pettazzoni P., Sacco E. (2015). 5-Fluorouracil resistant colon cancer cells are addicted to OXPHOS to survive and enhance stem-like traits. Oncotarget.

[9] Liberti M.V., Locasale J.W. (2016). The Warburg effect: How does it benefit cancer cells?. Trends Biochem. Sci..

[10] Owen O.E., Kalhan S.C., Hanson R.W. (2002). The key role of anaplerosis and cataplerosis for citric acid cycle function. J. Biol. Chem..

[11] Vander Heiden M.G., DeBerardinis R.J. (2017). Understanding the intersections between metabolism and cancer biology. Cell.

[12] Etchegaray J., Mostoslavsky R. (2016). Interplay between metabolism and epigenetics: A nuclear adaptation to environmental changes. Mol. Cell..

[13] Biswas S., Rao C.M. (2018). Epigenetic tools (the *Writers*, the *Readers* and the *Erasers*) and their implications in cancer therapy. Eur. J. Pharmacol..

[14] Kinnaird A., Zhao S., Wellen K.E., Michelakis E.D. (2016). Metabolic control of epigenetics in cancer. Nat. Rev. Cancer.

[15] Reid M.A., Dai Z., Locasale J.W. (2017). The impact of cellular metabolism on chromatin dynamics and epigenetics. Nat. Cell Biol..

[16] Campbell S.L., Wellen K.E. (2018). Metabolic signaling to the nucleus in cancer review metabolic signaling to the nucleus in cancer. Mol. Cell..

[17] Kim J., Yeom Y.I. (2018). Metabolic signaling to epigenetic alterations in cancer. Biomol Ther..

[18] Nebbioso A., Tambaro F.P., Aversana C.D., Altucci L. Cancer epigenetics: Moving forward. PLoS Genet..

[19] Sharma S., Kelly T.K., Jones P.A. (2010). Epigenetics in cancer. Carcinogenesis.

[20] Flavahan W.A., Gaskell E., Bernstein B.E. (2018). Epigenetic plasticity and the hallmarks of cancer. Science.

[21] Wong C.C., Qian Y., Yu J. (2017). Interplay between epigenetics and metabolism in oncogenesis: Mechanisms and therapeutic approaches. Oncogene.

[22] Miranda-Gonçalves V., Lameirinhas A., Henrique R., Jerónimo C. Metabolism and epigenetic interplay in cancer: Regulation and putative therapeutic targets. Front. Genet..

[23] Boukouris A.E., Zervopoulos S.D., Michelakis E.D. (2016). Metabolic enzymes moonlighting in the nucleus: Metabolic regulation of gene transcription. Trends Biochem. Sci..

[24] Zhao Z., Wang L., Di L. (2016). Compartmentation of metabolites in regulating epigenomes of cancer. Mol. Med..

[25] Newman A.C., Maddocks O.D.K. (2017). Serine and functional metabolites in cancer. Trends Cell Biol..

[26] Mattaini K.R., Sullivan M.R., Vander Heiden M.G. (2016). The importance of serine metabolism in cancer. J. Cell Biol..

[27] Amelio I., Cutruzzola F., Agostini M., Melino G. (2014). Serine and glycine metabolism in cancer. Trends Biochem. Sci..

[28] Locasale J.W. (2013). Serine, glycine and the one-carbon cycle: Cancer metabolism in full circle. Nat. Rev. Cancer.

[29] Luo J., Li Y., Wang F., Zhang W., Geng X. (2010). S-Adenosylmethionine inhibits the growth of cancer cells by reversing the hypomethylation status of c-myc and H-ras in human gastric cancer and colon cancer. Int. J. Biol. Sci..

[30] Ilisso C.P., Sapio L., Delle Cave D., Illiano M., Spina A., Cacciapuoti G., Naviglio S., Porcelli M. (2016). S-adenosylmethionine affects ERK1/2 and Stat3 pathways and induces apoptosis in osteosarcoma cells. J. Cell Physiol..

[31] Shukeir N., Stefanska B., Parashar S., Chik F., Arakelian A., Szyf M., Rabbani S.A. (2015). Pharmacological methyl group donors block skeletal metastasis in vitro and in vivo. Br. J. Pharmacol..

[32] Ilisso C.P., Delle Cave D., Mosca L., Pagano M., Coppola A., Mele L., Caraglia M., Cacciapuoti G., Porcelli M. (2018). S-Adenosylmethionine regulates apoptosis and autophagy in MCF-7 breast cancer cells through the modulation of specific microRNAs. Cancer Cell Int..

[33] Dai Z., Mentch S.J., Gao X., Nichenametla S.N., Locasale J.W. (2018). Methionine metabolism influences genomic architecture and gene expression through H3K4me3 peak width. Nat. Commun..

[34] Wellen K.E., Hatzivassiliou G., Sachdeva U.M., Bui T.V., Cross J.R., Thompson C.B. (2009). ATP-citrate lyase links cellular metabolism to histone acetylation. Science.

[35] Lee J.V., Carrer A., Shah S., Snyder N.W., Wei S., Venneti S., Worth A.J., Yuan Z.F., Lim H.W., Liu S.E. (2014). Akt-dependent metabolic reprogramming regulates tumor cell histone acetylation. Cell Metab..

[36] Pietrocola F., Galluzzi L., Bravo-San Pedro J.M., Madeo F., Kroemer G. (2015). Acetyl Coenzyme A: A central metabolite and second messenger. Cell Metab..

[37] Sivanand S., Viney I., Wellen K.E. (2018). Spatiotemporal control of acetyl-CoA metabolism in chromatin regulation. Trends Biochem. Sci..

[38] Mullen A.R., Wheaton W.W., Jin E.S., Chen P.H., Sullivan L.B., Cheng T., Yang Y., Linehan W.M., Chandel N.S., DeBerardinis R.J. (2012). Reductive carboxylation supports growth in tumour cells with defective mitochondria. Nature.

[39] McBrian M.A., Behbahan I.S., Ferrari R., Su T., Huang T.W., Li K., Hong C.S., Christofk H.R., Vogelauer M.D., Seligson B. (2013). Histone acetylation regulates intracellular pH. Mol. Cell..

[40] Erecińska M., Deas J., Silver I.A. (1995). The effect of pH on glycolysis and phosphofructokinase activity in cultured cells and synaptosomes. J. Neurochem..

[41] Shimazu T., Hirschey M.D., Newman J., He W., Shirakawa K., Le Moan N., Grueter C.A., Lim H., Saunders L.R., Stevens R.D. (2013). Suppression of oxidative stress by β-hydroxybutyrate, an endogenous histone deacetylase inhibitor. Science.

[42] Donohoe D.R., Collins L.B., Wali A., Bigler R., Sun W., Bultman S.J. (2012). The Warburg effect dictates the mechanism of butyrate mediated histone acetylation and cell proliferation. Mol. Cell..

[43] McDonough M.A., Loenarz C., Chowdhury R., Clifton I.J., Schofield C.J. (2010). Structural studies on human 2-oxoglutarate dependent oxygenases. Curr. Opin. Struct. Biol..

[44] Carey B.W., Finley L.W.S., Cross J.R., Allis C.D., Thompson C.B. (2015). Intracellular α-ketoglutarate maintains the pluripotency of embryonic stem cells. Nature.

[45] Van der Knaap J.A., Verrijzer C.P. (2016). Undercover: Gene control by metabolites and metabolic enzymes. Genes Dev..

[46] Pan M., Reid M.A., Lowman X.H., Kulkarni R.P., Tran T.Q., Liu X., Yang Y., Hernandez-Davies J.E., Rosales K.K., Li H. (2016). Regional glutamine deficiency in tumors promotes dedifferentiation through inhibition of histone demethylation. Nat. Cell Biol..

[47] Calvert A.E., Chalastanis A., Wu Y., Hurley L.A., Kouri F.M., Bi Y., Kachman M., May J.L., Bartom E., Hua Y. (2017). Cancer-associated IDH1 promotes growth and resistance to targeted therapies in the absence of mutation. Cell Rep..

[48] Schuettengruber B., Bourbon H.M., Di Croce L., Cavalli G. (2017). Genome regulation by Polycomb and Trithorax: 70 years and counting. Cell.

[49] Hwang I.Y., Kwak S., Lee S., Kim H., Lee S.E., Kim J.H., Kim Y.A., Jeon Y.K., Chung D.H., Jin X. (2016). Psat1-dependent fluctuations in α-ketoglutarate affect the timing of ESC differentiation. Cell Metab..

[50] Nagaoka K., Hino S., Sakamoto A., Anan K., Takase R., Umehara T., Yokoyama S., Sasaki Y., Nakao M. (2015). Lysine-specific demethylase 2 suppresses lipid influx and metabolism in hepatic cells. Mol. Cell Biol..

[51] Yang S.J., Park Y.S., Cho J.H., Moon B., An H.J., Lee J.Y., Xie Z., Wang Y., Pocalyko D., Lee D.C. (2017). Regulation of hypoxia responses by flavin adenine dinucleotide-dependent modulation of HIF-1α protein stability. EMBO J..

[52] Wojcieszyńska D., Hupert-Kocurek K., Guzik U. (2012). Flavin-dependent enzymes in cancer prevention. Int J. Mol. Sci..

[53] Amente S., Lania L., Majello B. (2013). The histone LSD1 demethylase in stemness and cancer transcription programs. Biochim. Biophys Acta.

[54] Collins R.R.J., Patel K., Putnam W.C., Kapur P., Rakheja D. (2017). Oncometabolites: A new paradigm for oncology, metabolism, and the clinical laboratory. Clin. Chem..

[55] Sciacovelli M., Frezza C. (2016). Oncometabolites: Unconventional triggers of oncogenic signal. Free Radic. Biol. Med..

[56] Skinner R., Trujillo A., Ma X., Beierle E.A. (2009). Ketone bodies inhibit the viability of human neuroblastoma cells. J. Pediatr. Surg..

[57] Shukla S.K., Gebregiworgis T., Purohit V., Chaika N.V., Gunda V., Radhakrishnan P., Mehla K., Pipinos I.I., Powers R., Yu F. (2014). Metabolic reprogramming induced by ketone bodies diminishes pancreatic cancer cachexia. Cancer Metab..

[58] Bonuccelli G., Tsirigos A., Whitaker-Menezes D., Pavlides S., Pestell R.G., Chiavarina B., Frank P.G., Flomenberg N., Howell A., Martinez-Outschoorn U.E. (2010). Ketones and lactate ‘fuel’ tumor growth and metastasis: Evidence that epithelial cancer cells use oxidative mitochondrial metabolism. Cell Cycle.

[59] Martinez-Outschoorn U.E., Lin Z., Whitaker-Menezes D., Howell A., Sotqia F., Lisanti M.P. (2012). Ketone body utilization drives tumor growth and metastasis. Cell Cycle.

[60] Rodrigues L.M., Uribe-Lewis S., Madhu B., Honess D.J., Stubbs M., Griffiths J.R. (2017). The action of β-hydroxybutyrate on the growth, metabolism and global histone H3 acetylation of spontaneous mouse mammary tumours: Evidence of a β-hydroxybutyrate paradox. Cancer Metab..

[61] Menendez J.A., Corominas-Faja B., Cujàs E., García M.G., Fernández-Arroyo S., Fernández A.F., Joven J., Fraga M.F., Alarcón T. (2016). Oncometabolic nuclear reprogramming of cancer stemness. Stem Cell Rep..

[62] Dando I., Pozza E.D., Ambrosini G., Torrens-Mas M., Butera G., Mullappilly N., Pacchiana R., Palmieri M., Donadelli M. (2019). Oncometabolites in cancer aggressiveness and tumour repopulation. Biol Rev. Camb. Philos. Soc..

[63] Condelli V., Crispo F., Pietrafesa M., Lettini G., Matassa D.S., Esposito F., Landriscina M., Maddalena F. (2019). HSP90 molecular chaperone, metabolic rewiring and epigenetics: Impact on tumor progression and perspective for anticancer therapy. Cells.

[64] Sciacovelli M., Guzzo G., Morello V., Frezza C., Zheng L., Nannini N., Calabrese F., Laudiero G., Esposito F., Landriscina M. (2013). The mitochondrial chaperone TRAP1 promotes neoplastic growth by inhibiting succinate dehydrogenase. Cell Metab..

[65] Lettini G., Maddalena F., Sisinni L., Condelli V., Matassa D.S., Costi M.P., Simoni D., Esposito F., Landriscina M. (2017). Targets TRAP1: A viable therapeutic target for future cancer treatments?. Expert Opin. Ther. Targets.

[66] Matassa D.S., Agliarulo I., Avolio R., Landriscina M., Esposito F. TRAP1 regulation of cancer metabolism: Dual role as oncogene or tumor suppressor. Genes.

[67] Sudarshan S., Shanmugasundaram K., Naylor S.L., Lin S., Livi C.B., O’Neill C.F., Parekh D.J., Yeh I.T., Sun L.Z., Block K. (2011). Reduced expression of fumarate hydratase in clear cell renal cancer mediates HIF-2α accumulation and promotes migration and invasion. PLoS ONE.

[68] Sciacovelli M., Gonçalves E., Johnson T.I., Zecchini V.R., Henriques da Costa A.S., Gaude E., Drubbel A.V., Theobald S.J., Abbo S., Tran M. (2016). Fumarate is an epigenetic modifier that elicits epithelial-to- mesenchymal transition. Nature.

[69] Intlekofer A.M., Dematteo R.G., Venneti S., Finley L.W.S., Lu C., Judkins A.R., Rustenburg A.S., Grinaway P.B., Chodera J.D., Cross J.R. (2015). Hypoxia induces production of L-2-hydroxyglutarate. Cell Metab..

[70] Intlekofer A.M., Wang B., Liu H., Shah H., Carmona-Fontaine C., Rustenburg A.S., Salah S., Gunner M.R., Chodera J.D., Cross J.R. (2017). L-2-hydroxyglutarate production arises from non-canonical enzyme function at acidic pH. Nat. Chem. Biol..

[71] Katada S., Imhof A., Sassone-Corsi P. (2012). Connecting threads: Epigenetics and metabolism. Cell.

[72] Nieborak A., Schneider R. (2018). Metabolic intermediates–cellular messangers talking to chromatin modifiers. Mol. Metab..

[73] Reytor E., Pérez-Miguelsanz J., Álvarez L., Pérez-Sala D., Pajares M.A. (2009). Conformational signals in the C-terminal domain of methionine adenosyltransferase I/III determine its nucleocytoplasmic distribution. FASEB J..

[74] Pajares M.A., Álvarez L., Pérez-Sala D. (2013). How are mammalian methionine adenosyltransferases regulated in the liver? A focus on redox stress. FEBS Lett..

[75] Katoh Y., Ikura T., Hoshikawa Y., Tashiro S., Ito T., Ohta M., Kera Y., Noda T., Igarashi K. (2011). Methionine adenosyltransferase II serves as a transcriptional corepressor of Maf oncoprotein. Mol. Cell..

[76] Kera Y., Katoh Y., Ohta M., Matsumoto M., Takano-Yamamoto T., Igarashi K. (2013). Methionine adenosyltransferase II-dependent histone H3K9 methylation at the COX-2 gene locus. J. Biol Chem..

[77] Li S., Gogol S.K., Florens L., Washburn M.P., Jerry L. (2015). Serine and SAM responsive complex SESAME regulates histone modification crosstalk by sensing cellular metabolism. Mol. Cell.

[78] Schug Z.T., Peck B., Jones D.T., Zhang Q., Grosskurth S., Alam I.S., Goodwin L.M., Smethurst E., Mason S., Blyth K. (2015). Acetyl-CoA synthetase 2 promotes acetate utilization and maintains cancer cell growth under metabolic stress. Cancer Cell.

[79] Bulusu V., Tumanov S., Michalopoulou E., van den Broek N.J., MacKay G., Nixon C., Dhayade S., Schug Z.T., Vande Voorde J., Blyth K. (2017). Acetate recapturing by nuclear acetyl-CoA synthetase 2 prevents loss of histone acetylation during oxygen and serum limitation. Cell Rep..

[80] Takahashi H., McCaffery M., Irizarry R.A., Boeke J.D. (2006). Nucleocytosolic acetyl-coenzyme A synthetase is required for histone acetylation and global transcription. Mol. Cell.

[81] Li X., Yu W., Qian X., Xia Y., Zheng Y., Lee J.H., Li W., Lyu J., Rao G., Zhang X. (2017). Nucleus-translocated ACSS2 promotes gene transcription for lysosomal biogenesis and autophagy. Mol. Cell.

[82] Sivanand S., Rhoades S., Jiang Q., Lee J.V., Benci J., Zhang J., Yuan S., Viney I., Zhao S., Carrer A. (2017). Nuclear acetyl-CoA production by ACLY promotes homologous recombination. Mol. Cell.

[83] Sutendra G., Kinnaird A., Dromparis P., Paulin R., Stenson T.H., Haromy A., Hashimoto K., Zhang N., Flaim E., Michelakis E.D. (2014). A nuclear pyruvate dehydrogenase complex is important for the generation of acetyl-CoA and histone acetylation. Cell.

[84] Chen J., Guccini I., Mitri D.D., Brina D., Revandkar A., Sarti M., Pasquini E., Alajati A., Pinton S., Losa M. (2018). Compartmentalized activities of the pyruvate dehydrogenase complex sustain lipogenesis in prostate cancer. Nat. Genet..

[85] Madiraju P., Pande S.V., Prentki M., Murthy Madiraju S.R. (2009). Mitochondrial acetylcarnitine provides acetyl groups for nuclear histone acetylation. Epigenetics.

[86] Yang W., Lu Z. (2013). Nuclear PKM2 regulates the Warburg effect. Cell Cycle.

[87] Desai S., Ding M., Wang B., Lu Z., Zhao Q., Shaw K., Yung W.K.A., Weinstein J.N., Tan M., Yao J. (2014). Tissue-specific isoform switch and DNA hypomethylation of the pyruvate kinase PKM gene in human cancers. Oncotarget.

[88] Yang W., Xia Y., Ji H., Zheng Y., Liang J., Huang W., Gao X., Aldape K., Lu Z. (2011). Nuclear PKM2 regulates β-catenin transactivation upon EGFR activation. Nature.

[89] Yang W., Xia Y., Hawke D., Li X., Liang J., Xing D., Aldape K., Hunter T., Yung W.K.A., Lu Z. (2012). PKM2 phosphorilates histone H3 and promotes gene transcription and tumorigenesis. Cell.

[90] Wang H.J., Hsieh Y.J., Cheng W.C., Lin C.P., Lin Y.S., Yang S.F., Chen C.C., Izumiya Y., Yu J.S., Kung H.J. (2014). JMJD5 regulates PKM2 nuclear translocation and reprograms HIF-1α–mediated glucose metabolism. Proc. Natl. Acad. Sci. USA.

[91] Matsuda S., Adachi J., Ihara M., Tanuma N., Shima H., Kakizuka A., Ikura M., Ikura T., Matsuda T. (2016). Nuclear pyruvate kinase M2 complex serves as a transcriptional coactivator of aryl hydrocarbon receptor. Nucleic Acids Res..

[92] Minchenko O., Opentanova I., Minchenko D., Ogura T., Esumi H. (2004). Hypoxia induces transcription of 6-phosphofructo-2-kinase/fructose-2,6-biphosphatase-4 gene via hypoxia-inducible factor-1α activation. FEBS Lett..

[93] Chesney J., Lane A.N. (2014). Fructose-2,6-bisphosphate synthesis by required for the glycolytic response to hypoxia and tumor growth. Oncotarget.

[94] Dasgupta S., Rajapakshe K., Zhu B., Nikolai B.C., Yi P., Putluri N., Choi J.M., Jung S.Y., Coarfa C., Westbrook T.F. (2018). Metabolic enzyme PFKFB4 activates transcriptional coactivator SRC-3 to drive breast cancer. Nature.

[95] Castonguay Z., Auger C., Thomas S.C., Chahma M., Appanna V.D. (2014). Nuclear lactate dehydrogenase modulates histone modification in human hepatocytes. Biochem. Biophys Res. Commun..

[96] Zhang J.Y., Zhang F., Hong C.Q., Giuliano A.E., Cui X.J., Zhou G.J., Zhang G.J., Cui Y.K. (2015). Critical protein GAPDH and its regulatory mechanisms in cancer cells. Cancer Biol. Med..

[97] Hara M.R., Agrawal N., Kim S.F., Cascio M.B., Fujimuro M., Ozeki Y., Takahashi M., Cheah J.H., Tankou S.K., Hester L.D. (2005). S-nitrosylated GAPDH initiates apoptotic cell death by nuclear translocation following Siah1 binding. Nat. Cell Biol..

[98] Kornberg M.D., Sen N., Hara M.R., Juluri K.R., Nguyen J.V.K., Snowman A.M., Law L., Hester L.D., Snyder S.H. (2010). GAPDH mediates nitrosylation of nuclear proteins. Nat. Cell Biol..

[99] Monaghan R.M., Whitmarsh A.J. (2015). Mitochondrial proteins moonlighting in the nucleus. Trends Biochem Sci..

[100] Jiang Y., Qian X., Shen J., Wang Y., Li X., Liu R., Xia Y., Chen Q., Peng G., Lin S.Y. (2015). Local generation of fumarate promotes DNA repair through inhibition of histone H3 demethylation. Nat. Cell Biol..

[101] May J.L., Kouri F.M., Hurley L.A., Liu J., Tommasini-Ghelfi S., Ji Y., Gao P., Calvert A.E., Lee A., Chandel N.S. (2019). IDH3α regulates one-carbon metabolism in glioblastoma. Sci. Adv..

[102] Agarwal S., Sharma M.C., Jha P., Pathak P., Suri V., Sarkar C., Chosdol K., Suri A., Kale S.S., Mahapatra A.K. (2013). Comparative study of IDH1 mutations in gliomas by immunohistochemistry and DNA sequencing. Neuro. Oncol..

[103] Ying W. (2008). NAD^+^/NADH and NADP^+^/NADPH in cellular functions and cell death: Regulation and biological consequences. Antioxid Redox Signal..

[104] Goel A., Mathupala S.P., Pedersen P.L. (2003). Glucose metabolism in cancer. Evidence that demethylation events play a role in activating type II hexokinase gene expression. J. Biol. Chem..

[105] Lee H.G., Kim H., Son T., Jeong Y., Kim S.U., Dong S.M., Park Y.N., Lee J.D., Lee J.M., Park J.H. (2016). Regulation of HK2 expression through alterations in CpG methylation of the HK2 promoter during progression of hepatocellular carcinoma. Oncotarget.

[106] Wolf A., Agnihotri S., Munoz D., Guha A. (2011). Neurobiology of disease developmental profile and regulation of the glycolytic enzyme hexokinase 2 in normal brain and glioblastoma multiforme. Neurobiol. Dis..

[107] Dong Z., Cui H. (2018). Epigenetic modulation of metabolism in glioblastoma. Semin. Cancer Biol..

[108] Chen M., Zhang J., Li N., Qian Z., Zhu M., Li Q., Zheng J., Wang X., Shi G. (2011). Promoter hypermethylation mediated downregulation of FBP1 in human hepatocellular carcinoma and colon cancer. PLoS ONE.

[109] Dong C., Yuan T., Wu Y., Wang Y., Fan T.W.M., Miriyala S., Lin Y., Yao J., Shi J., Kang T. (2013). Loss of FBP1 by Snail-mediated repression provides metabolic advantages in basal-like breast cancer. Cancer Cell.

[110] Li L., Li W. (2015). Epithelial–mesenchymal transition in human cancer: Comprehensive reprogramming of metabolism, epigenetics, and differentiation. Pharmacol. Ther..

[111] Pan D., Mao C., Wang Y. (2013). Suppression of gluconeogenic gene expression by LSD1-mediated histone demethylation. PLoS ONE.

[112] Lopez-Serra P., Marcilla M., Villanueva A., Ramos-Fernandez A., Palau A., Leal L., Wahi J.E., Setien-Baranda F., Szczesna K., Moutinho C. (2014). A DERL3-associated defect in the degradation of SLC2A1 mediates the Warburg effect. Nat. Commun..

[113] Salvesen H.B., MacDonald N., Ryan A., Jacobs I.J., Lynch E.D., Akslen L.A., Das S. (2001). PTEN methylation is associated with advanced stage and microsatellite instability in endometrail carcinoma. Int. J. Cancer.

[114] Kang Y., Lee H.S., Kim W.H. (2002). Promoter methylation and silencing of PTEN in gastric carcinoma. Lab. Invest..

[115] Soria J.C., Lee H.Y., Lee J.I., Wang L., Issa J.P., Kemp B.L., Liu D.D., Kurie J.M., Mao L., Khuri F.R. (2002). Lack of PTEN expression in non-small cell lung cancer could be related to promoter methylation 1. Clin. Cancer Res..

[116] García J.M., Silva J., Peña C., Garcia V., Rodríguez R., Cruz M.A., Cantos B., Provencio M., España P., Bonilla F. (2004). Promoter methylation of the PTEN gene is a common molecular change in breast cancer. Genes Chromosom. Cancer.

[117] Alvarez-Nuñez F., Bussaglia E., Mauricio D., Ybarra J., Vilar M., Lerma E., de Leiva A., Matias-Guiu X. (2006). PTEN promoter methylation in sporadic thyroid carcinomas. Thyroid.

[118] Trojan J., Brieger A., Raedle J., Esteller M., Zeuzem S. (2000). 5′-CpG island methylation of the LKB1/STK11 promoter and allelic loss at chromosome 19p13.3 in sporadic colorectal cancer. Gut.

[119] Esteller M., Avizienyte E., Corn P.G., Lothe R.A., Baylin S.B., Aaltonen L.A., Herman J.G. (2000). Epigenetic inactivation of LKB1 in primary tumors associated with the Peutz-Jeghers syndrome. Oncogene.

[120] Herman J.G., Latif F., Weng Y., Lerman M.I., Zbar B., Liu S., Samid D., Duan D.S.R., Gnarra J.R., Linehan W.M. (1994). Silencing of the VHL tumor-suppressor gene by DNA methylation in renal carcinoma. Proc. Natl. Acad. Sci. USA.

[121] Schmitt A.M., Schmid S., Rudolph T., Anlauf M., Prinz C., Klöppel G., Moch H., Heitz P.U., Komminoth P., Perren A. (2009). VHL inactivation is an important pathway for the development of malignant sporadic pancreatic endocrine tumors. Endocr. Relat. Cancer.

[122] Vanharanta S., Shu W., Brenet F., Hakimi A.A., Heguy A., Viale A., Reuter V.E., Hsieh J.J.D., Scandura J.M., Massagué J. (2013). Epigenetic expansion of VHL-HIF signal output drives multiorgan metastasis in renal cancer. Nat. Med..

[123] Place T.L., Fitzgerald M.P., Venkataraman S., Vorrink S.U., Case A.J., Teoh M.L., Domann F.E. (2011). Aberrant promoter CpG methylation is a mechanism for impaired PHD3 expression in a diverse set of malignant cells. PLoS ONE.

[124] Rawluszko A., Bujnicka K.B., Horbacka K., Krokowicz P., Jagodziński P.P. (2013). Expression and DNA methylation levels of prolyl hydroxylase PHD1, PHD2, PHD3 and asparaginyl hydroxylase FIH in colorectal cancer. BMC Cancer.

[125] Wan W., Peng K., Li M., Qin L., Tong Z., Yan J., Shen B., Yu C. (2017). Histone demethylase JMJD1A promotes urinary bladder cancer progression by enhancing glycolysis through coactivation of hypoxia inducible factor 1α. Oncogene.

[126] Luo L., Chang R., Zhong J., Pandey A., Semenza G.L. (2012). Histone demethylase JMJD2C is a coactivator for hypoxia-inducible factor 1 that is required for breast cancer progression. Proc. Natl. Acad. Sci. USA.

[127] Kozono D., Li J., Nitta M., Sampetrean O., Gonda D., Kushwaha D.S., Merzon D., Ramakrishnan V., Zhu S., Zhu K. (2015). Dynamic epigenetic regulation of glioblastoma tumorigenicity through LSD1 modulation of MYC expression. Proc. Natl. Acad. Sci. USA.

[128] Chalkiadaki A., Guarente L. (2015). The multifaceted functions of sirtuins in cancer. Nat. Rev. Cancer.

[129] Sebastián C., Zwaans B.M.M., Silberman D.M., Gymrek M., Goren A., Zhong L., Ram O., Truelove J., Guimaraes A.R., Toiber D. (2012). The histone deacetylase SIRT6 is a tumor suppressor that controls cancer metabolism. Cell.

[130] Hallows W.C., Yu W., Denu J.M. (2012). Regulation of glycolytic enzyme phosphoglycerate mutase-1. J. Biol. Chem..

[131] Liu P.Y., Xu N., Malyukova A., Scarlett C.J., Sun Y.T., Zhang X.D., Ling D., Su S.P., Nelson C., Chang D.K. (2013). The histone deacetylase SIRT2 stabilizes Myc oncoproteins. Cell Death Diff..

[132] Matilainen O., Quirós P.M., Auwerx J. (2017). Mitochondria and epigenetics–crosstalk in homeostasis and stress. Trends Cell Biol..

[133] Rinaldi G., Rossi M., Fendt S.M. Metabolic interactions in cancer: Cellular metabolism at the interface between the microenvironment, the cancer cell phenotype and the epigenetic landscape. Wiley Interdiscip. Rev. Syst. Biol. Med..

[134] Domblides C., Lartigue L., Faustin B. (2019). Control of the antitumor immune response by cancer metabolism. Cells.

[135] Wegiel B., Vuerich M., Daneshmandi S., Seth P. (2018). Metabolic switch in the tumor microenvironment determines immune responses to anti-cancer therapy. Front. Oncol..

[136] Colegio O.R., Chu N.-Q., Szabo A.L., Chu T., Rhebergen A.M., Jairam V., Cyrus N., Brokowski C.E., Eisenbarth S.C., Phillips G.M. (2014). Functional polarization of tumour-associated macrophages by tumour-derived lactic acid. Nature.

[137] Ippolito L., Morandi A., Giannoni E., Chiarugi P. (2019). Lactate: A metabolic driver in the tumour landscape. Trends Biochem. Sci..

[138] Mu X., Shi W., Xu Y., Xu C., Zhao T., Geng B., Yang J., Pan J., Hu S., Zhang C. (2018). Tumor-derived lactate induces M2 macrophage polarization via the activation of the ERK/STAT3 signaling pathway in breast cancer. Cell Cycle.

[139] Laoui D., Van Overmeire E., Di Conza G., Aldeni C., Keirsse J., Morias Y., Movahedi K., Houbracken I., Schouppe E., Elkrim Y. (2014). Tumor hypoxia does not drive differentiation of tumor-associated macrophages but rather fine-tunes the M2-like macrophage population. Cancer Res..

[140] Carmona-Fontaine C., Deforet M., Akkari L., Thompson C.B., Joyce J.A., Xavier J.B. (2017). Metabolic origins of spatial organization in the tumor microenvironment. Proc. Natl. Acad. Sci. USA..

[141] Seth P., Csizmadia E., Hedblom A., Vuerich M., Xie H., Li M., Longhi M.S., Wegiel B. (2017). Deletion of Lactate Dehydrogenase-A in Myeloid Cells Triggers Antitumor Immunity. Cancer Res..

[142] Renner K., Singer K., Koehl G.E., Geissler E.K., Peter K., Siska P.J., Kreutz M. (2017). Metabolic hallmarks of tumor and immune cells in the tumor microenvironment. Front. Immunol..

[143] Kouidhi S., Ben Ayed F., Benammar Elgaaied A. (2018). Targeting Tumor Metabolism: A New Challenge to Improve Immunotherapy. Front. Immunol..

[144] Mocellin S., Wang E., Marincola F.M. (1991). Cytokines and immune response in the tumor microenvironment. J. Immunother..

[145] Vega M.A., Corbí A.L. (2006). Human macrophage activation: Too many functions and phenotypes for a single cell type. Inmunología.

[146] Tekpli X., Landvik N.E., Anmarkud K.H., Skaug V., Haugen A., Zienolddiny S. (2013). DNA methylation at promoter regions of interleukin 1B, interleukin 6, and interleukin 8 in non-small cell lung cancer. Cancer Immunol. Immunother..

[147] Marks D.L., Olson R.L., Fernandez-Zapico M.E. (2016). Epigenetic control of the tumor microenvironment. Epigenomics..

[148] Latham T., Mackay L., Sproul D., Karim M., Culley J., Harrison D.J., Hayward L., Langridge-Smith P., Gilbert N., Ramsahoye B.H. (2012). Lactate, a product of glycolytic metabolism, inhibits histone deacetylase activity and promotes changes in gene expression. Nucleic Acids Res..

[149] Tran T.Q., Lowman X.H., Kong M. (2017). Molecular pathways: Metabolic control of histone methylation and gene expression in cancer. Clin. Cancer Res..

[150] Glazer R.I., Hartman K.D., Knode M.C., Richard M.M., Chiang P.K., Tseng C.K., Marquez V.E. (1986). 3-Deazaneplanocin: A new and potent inhibitor of s-adenosylhomocysteine hydrolase and its effects on human promyelocytic leukemia cell line HL-60. Biochem Biophys Res. Commun..

[151] Momparler R.L., Côté S., Momparler L.F., Idaghdour Y. Epigenetic therapy of acute myeloid leukemia using 5-aza-2′-deoxycytidine (decitabine) in combination with inhibitors of histone methylation and deacetylation. Clin. Epigenetics..

[152] Momparler R.L., Côté S. (2015). Targeting of cancer stem cells by inhibitors of DNA and histone methylation. Expert Opin. Investig. Drugs..

[153] Dhillon S. (2018). Ivosidenib: First global approval. Drugs.

[154] Yen K., Travins J., Wang F., David M.D., Artin E., Straley K., Padyana A., Gross S., De La Barre B., Tobin E. (2017). AG-221, a first-in-class therapy targeting acute myeloid leukemia harboring oncogenic idh2 mutations. Cancer Discov..

[155] Liu X.S., Little J.B., Yuan Z.M. (2015). Glycolytic metabolism influences global chromatin structure. Oncotarget.

[156] Moussaieff A., Rouleau M., Kitsberg D., Cohen M., Levy G., Barasch D., Nemirovski A., Shen-Orr S., Laevsky I., Amit M. (2015). Glycolysis-mediated changes in acetyl-CoA and histone acetylation control the early differentiation of embryonic stem cells. Cell Metab..

[157] Robinson M.M., McBryant S.J., Tsukamoto T., Rojas C., Ferraris D.V., Hamilton S.K. (2007). Novel mechanism of inhibition of rat kidney-type glutaminase by bis-2-(5- phenylacetamido-1,2,4-thiadiazol-2-yl)ethyl sulfide (BPTES). Biochem. J..

[158] Simpson N.E., Tryndyak V.P., Pogribna M., Beland F.A., Pogribny I.P. (2012). Modifying metabolically sensitive histone marks by inhibiting glutamine metabolism affects gene expression and alters cancer cell phenotype. Epigenetics.

[159] Elhammali A., Ippolito J.E., Collins L., Crowley J., Marasa J., Piwnica-Worms D. (2014). A high-throughput fluorimetric assay for 2-hydroxyglutarate identifies zaprinast as a glutaminase inhibitor. Cancer Discov..

[160] Ferrari A., Longo R., Silva R., Mitro N., Caruso D., De Fabiani E., Crestani M. (2018). Epigenome modifiers and metabolic rewiring: New frontiers in therapeutics. Pharmacol. Ther..

[161] Alcarraz-Vizán G., Boren J., Lee W.P., Cascante M. (2010). Histone deacetylase inhibition results in a common metabolic profile associated with HT29 differentiation. Metabolomics.

[162] Borodovsky A., Salmasi V., Turcan S., Fabius A.W., Baia G.S., Eberhart C.G., Weingart J.D., Gallia G.L., Baylin S.B., Chan T.A. (2013). 5-azacytidine reduces methylation, promotes differentiation and induces tumor regression in a patient-derived IDH1 mutant glioma xenograft. Oncotarget.

[163] Turcan S., Fabius A.W., Borodovsky A., Pedraza A., Brennan C., Huse J., Viale A., Riggins G.J., Chan T.A. (2013). Efficient induction of differentiation and growth inhibition in IDH1 mutant glioma cells by the DNMT Inhibitor Decitabine. Oncotarget.

[164] Fouad Y.A., Aanei C. (2017). Revisiting the hallmarks of cancer. Am. J. Cancer Res..

[165] Wardell S.E., Ilkayeva O.R., Wieman H.L., Frigo D.E., Rathmell J.C., Newgard C.B., McDonnell D.P. (2009). Glucose metabolism as a target of histone. Mol. Endocrinol..

[166] Amoêdo N.D., Rodrigues M.F., Pezzuto P., Galina A., da Costa R.M., de Almeida F.C., El-Bacha T., Rumjanek F.D. (2011). Energy metabolism in H460 lung cancer cells: Effects of histone deacetylase inhibitors. PLoS ONE.

[167] Rodrigues M.F., Carvalho É., Pezzuto P., Rumjanek F.D., Amoêdo N.D. (2015). Reciprocal modulation of histone deacetylase inhibitors sodium butyrate and trichostatin A on the energy metabolism of breast cancer cells. J. Cell Biochem..

[168] Mahmood N., Cheishvili D., Arakelian A., Tanvir I., Khan H.A., Pépin A.S., Szyf M., Rabbani S.A. (2018). Methyl donor S-adenosylmethionine (SAM) supplementation attenuates breast cancer growth, invasion, and metastasis in vivo; therapeutic and chemopreventive applications. Oncotarget.

[169] Li L., Paz A.C., Wilky B.A., Johnson B., Galoian K., Rosenberg A., Hu G., Tinoco G., Bodamer O., Trent J.C. (2015). Treatment with a small molecule mutant IDH1 inhibitor suppresses tumorigenic activity and decreases production of the oncometabolite 2-hydroxyglutarate in human chondrosarcoma cells. PLoS ONE.

[170] Dalle I.A., DiNardo C.D. (2018). The role of enasidenib in the treatment of mutant IDH2 acute myeloid leukemia. Ther. Adv. Hematol..

[171] Miranda T.B., Cortez C.C., Yoo C.B., Liang G., Abe M., Kelly T.K., Marquez V.E., Jones P.A. (2009). DZNep is a global histone methylation inhibitor that reactivates developmental genes not silenced by DNA methylation. Mol. Cancer Ther..

[172] Lin S.H., Liu T., Ming X., Tang Z., Fu L., Schmitt-Kopplin P., Kanawati B., Guan X.Y., Cai Z. Regulatory role of hexosamine biosynthetic pathway on hepatic cancer stem cell marker CD133 under low glucose conditions. Sci. Rep..

[173] Fiskus W., Wang Y., Sreekumar A., Buckley K.M., Shi H., Jillella A., Ustun C., Rao R., Fernandez P., Chen J. (2009). Combined epigenetic therapy with the histone methyltransferase EZH2 inhibitor 3-deazaneplanocin A and the histone deacetylase inhibitor panobinostat against human AML cells. Blood.

[174] Egler V., Korur S., Failly M., Boulay J., Imber R., Lino M.M., Merlo A. (2008). Histone deacetylase inhibition and blockade of the glycolytic pathway synergistically induce glioblastoma cell death. Cancer Ther. Preclin..

